# This Journal Is Yours

**Published:** 1916-05

**Authors:** 


### This Journal is Yours.

The editor of the TEXAS MEDICAL JOURNAL would like to have its every reader feel a close relationship with it. Great care and attention is given every number to the end that it may really be worth while; that it may be just what you would want it to be. Of course, everything in it does not interest every reader, but everyone should find something of real value in every issue. If you do not, the editor feels that something must be wrong with the JOURNAL. It will only cost you a stamp or a postcard to let the editor know wherein it fails. Will you not do this and thus help to make it better? It may be that your viewpoint and the editor's are not at all alike as to what a medical journal should be, and if it is and you have any helpful suggestions to offer they will be adopted if found practical. There is only one way to make any journal what it should be and that is by co-operation of the editor and the readers. Many readers write that the TEXAS MEDICAL JOURNAL is improving with every issue, but hundreds never say whether they like it or not. If you do not have the time to write about it and you think favorably of it, will you not do the publisher the favor to mention it to your brother practitioners, and in that way assist in increasing its circulation?

